# University College, Bristol. Medical School

**Published:** 1883-12

**Authors:** 


### UNIVERSITY COLLEGE, BRISTOL.

### MEDICAL SCHOOL.

THE Annual Distribution of Prizes took place on July 23rd, 1883, in the Theatre of the Bristol Museum and Library. In their Report the Faculty of the School stated that they had much pleasure in again drawing attention to the satisfactory condition of the School as regards both the number of their students and the excellent character of the work done during the year. The number of entirely new students had been 38, as against 27 in the previous year, and the total number of students on the books during the year was 90.

Dr. BEDDOE, F.R.S., who distributed the prizes, gave the following address:—

Is it possible to be original on an occasion such as the present? Scarcely possible, I think, in the sense of saying what is suitable as well as new, of saying what has never been said before on some similar occasion. Medical schools are many, and in all of them, year by year, come round the introductory lecture and the prize-day address. Fortunately the audience is always, to some extent at least, a new one. "The theory may be gray, but green is the golden tree of life." The prospect of the garden of science is ever new to those who are but just entering the gate, and among the new things to be contemplated is the history of the science and art of medicine. I am not going to ask your attention to the authenticated beginnings of this history in classic days, when gigantic forms such as those of Hippocrates and Aristotle moved through chaos and darkness, with little more of a fixed point to start from than Archimedes had for his lever. They were giants indeed; they threw penetrating glances to a great distance through that darkness, and fashioned out of the chaos forms that endure and have their value even in our days. But what I wish to consider is not so much the origin and progress, the flux and reflux (for it has not always been real progress), of medical knowledge and skill, as the history and development of the profession. Coleridge said there had been three silent revolutions—the first when the professions fell off from the Church; the second when literature fell off from the professions; and the third when the press fell off from literature. All these would be said, in the present day, to have occurred in the course of evolution by differentiation. The implication, however, that the votaries of science were in the dark ages all embraced within the limits of the clerical body, requires some qualification. Doubtless it is true that down almost to the days of Erasmus there was hardly room, outside the Church or the cloister, for any student not richly gifted with rank and wealth or personal prowess; and that, whether he sought the knowledge of law, or physic, or metaphysic, of man's relations to his fellow-man, or to nature, or to the supernatural. So in the days when bishops were jurists, men like the first great Bacon were monks and friars. But our profession owns a double or a triple ancestry; it is not only differentiation that has been at work in it during the last few centuries; there has also been a process of redintegration, of composition from diverse elements. The Anglo-Saxon—I beg Mr. Freeman's pardon—the English leech was not always a cleric; the physician of the Mediterranean lands was usually a Jew, or a wandering Greek, or even a Moslem. He may have been the lineal descendant of those precariously-existing vagabonds who are shadowed forth in old semi-mythical stories as existing among the Celts and their neighbours, notably among the Irish, where we hear of a royal invalid ornamenting the circuit of his palace with the severed heads, impaled on the national pike, of unsuccessful practitioners of medicine. The Jews inherited the secrets of their own tribe, and acquired in travel and commerce the knowledge of the Arabian and Persian mediciners. The frequent accusations of poisoning which were brought against them derived, doubtless, a colour from their knowledge of the use of drugs. But it was during the renaissance of learning that the separation of the profession from the Church, which Coleridge speaks of, became distinct and strongly marked. The physician—the physicist, as we now, with a barbarous cacophony, style him,—the student, the worshipper, the interrogator, the interpreter of Nature, was now comparatively seldom a monk, and still seldomer a priest. Still it was not until the Reformation that the final separation took place; and it is noteworthy that when the College of Physicians was constituted, two of its founders, and those the most eminent, viz., Chambre and Linacre, were in Holy Orders, the former having received ordination at an early age, the latter after having acquired reputation in medicine. On the other hand, Sir William Butts, physician to Henry the Eighth, and one of the earliest admitted Fellows of the College, appears in effigy on his tomb clad in the full armour of a knight. Dr. Master, the celebrated physician of Queen Elizabeth, to whom she gave the beautiful manor and house of Knole, near this city, was in Orders; but his appears to have been a case of simple change of profession, such as might occur in our own day. All these were doctors of medicine, of some British or foreign University, not of necessity, but in compliance with a custom, which has continued almost universal among British physicians up to the present time. Hence it is that in England this word doctor, indicating properly, as you are aware, the possession of a University degree, it may be in theology, in laws, in medicine, in philosophy, or even in music, is customarily but erroneously used to denote a medical practitioner; while the good old Saxon word "leech," once so convenient for that purpose, is enjoyed by a certain little animal less familiar indeed to the present than to the past generation, but supposed to be potent in healing.

The profession of the medical physician, we have seen, was regularly and separately constituted more than three centuries ago, and has undergone since then little formal change. But the ranks of the artists in healing were to be recruited from two other sources, the surgeons and the apothecaries; the former originally regarded as artizans, the latter as traders. The small repute in which surgery used to be held, not only in Europe but in other half-civilised countries, is very remarkable and not altogether easy of explanation. In our own day, undoubtedly, the brilliant triumphs of surgery appeal much more forcibly to the uneducated critic, if not to the educated one, than the more obscure and doubtful victories of medicine. Perhaps in old times the mysteriousness of medicine imposed more upon the imagination; at all events, it was practised by men intellectually and socially superior to the rough, ignorant fellows to whom surgery was abandoned. The best thing about them was their good old Greek name, χειρουργός—the hand-worker, the handy-man, the proper man of his hands. But to the German the surgeon was a Wundarzt, an artist or artisan who had to do with wounds: and, nowadays, these same Germans use the word Arzt (artist) pretty much as the commonalty among us use that of doctor—regarding the healing art, apparently, as the highest and most useful of arts, the art *par excellence;* whereas we have labelled it as a science taught and put to practical use. There was indeed something to be said in favour of this separation of the two great branches of the healing art; a separation not peculiar to this stage of western civilization, but equally consonant with Oriental ideas. The Ambastha, or physician caste, has always been highly honoured in India, but positively forbidden to practise certain surgical operations, which are left to low-caste men. In more modern times many great intellects have been devoted to surgery. I apprehend that such men as Astley Cooper and Nélaton, while they equalled any of their purely medical contemporaries in repute, surpassed them in revenue; and we have seen Sir James Paget in the chair of the greatest International Medical Congress that has ever assembled. Yet, certainly, there is an important difference between the medical and the surgical intellect in their most typical examples, and it was indicated in the sneering expression said to have been used by one of the greatest of surgeons, Syme, with reference to an eminent rival:—"A very clever carpenter," he said. A good operator and a great engineer are made out of nearly the same stuff, while the physician is more akin to the philosopher or the natural historian. Public convenience makes it almost necessary that medicine and surgery should be generally practised in conjunction, at all events in thinly-peopled districts, where, indeed, the function of the apothecary is usually, and almost of necessity, superadded ; but in great cities it is probably better that they should be carried on separately. Only an Admirable Crichton could attain and maintain a high degree of skill in all branches of the profession. Whether any, and what, further subdivisions of practice should be made, in communities sufficiently large and wealthy to make them pay, is a matter of detail into which I do not propose to enter, albeit it is not remotely connected with the subject I am next going to touch upon.

One often hears it said that the present is an era of competitive examinations. Those who use the expression may be logically taken to be in some measure opponents of the system ; to entertain, at least, suspicions that it is carried too far ; that it will not continue for ever, but must and will be abated in time to come. Pass-examinations are thought less objectionable. Scarcely anyone, I believe, has had the hardihood to apply to physic the principles of free trade and of "*caveat emptor,*" and to propose the abolition of all examination tests that guard the entrance to the profession. Anyone whom it pleases may indeed attempt to practise the healing art ; and there are few of us, I trust, who would seek to restrict the liberty of the credulous to undervalue their own lives and health, and to entrust them to the keeping of charlatans. But the State takes some care that at least they shall do this with their eyes open ; and that such charlatans shall not counterfeit the Government stamp which is impressed on those who have complied with its conditions, the chief of which is the passing of certain examinations, framed by some sixteen or seventeen learned bodies, to whom the matter has been entrusted. There is a wide-spread feeling, among those who have themselves no more examinations to pass, in favour of rendering the pass-examinations both more stringent and more uniform. But perhaps something may be said on the other side. There is no one hard and fast line which would not occasionally shut out a valuable man, while it would include some who would prove stupid and incompetent. If we wish to foster and give fair-play to the original and varied types of mind which abound in England, we should not attempt to run them all into exactly the same mould. No school, no curriculum, should be allowed to be weak in any department; but there might be no harm if a little more stress were laid, here on one class of subject, there on another, liberty of migration being allowed and made easy to students during the latter part of their curriculum. The correlative of too stringent examinations is excessive grinding, a process apt to be very injurious to the mind of the student. They also lead to an undue prolongation of the pupilage. Whatever the number of years allotted for medical education, it is of great importance that the last of them should be occupied in honest and untrammelled efforts to acquire, in hospitals or dispensaries, a practical knowledge of medical work; and men who are worth anything will do this far more freely, vigorously and effectually if they are not bound down in a particular groove by the requirements of some impending examination. Attendance and work should, I believe, be exacted at that period, rather than the performance of a certain specified minimum of paper-work or *vivâ-voce* answering. There is, moreover, one important requirement to be fulfilled by those, who, like the Convocation of a certain University of which I am a graduate, seem to think that every mortal defect can be cured, and can only be cured, by more and more stringent examinations. Infallible examiners, or something approaching to infallible ones, must be provided. Now experience leads many of us to think that most examiners are far, very far indeed, from being infallible. It would appear that some people think that the possession of extensive and accurate knowledge on the subject of examination is the only necessary qualification for an examiner. This is a great mistake. The possession of knowledge no more enables a man to *examine* than it qualifies him to *teach*. Not very long ago I received from Cambridge the proposals for the foundation of a scholarship in scientific research, in memory of the lamented Professor Balfour. And I observed that among the few canons laid down respecting such scholarship was the following:—"The holder of this Scholarship shall not be elected by competitive examination." "We in Cambridge," said Michael Foster, "are thought to have carried the art of examination to a high pitch of perfection, so high, indeed, that in trying to get hold of the best men we are usually successful in getting hold of the second best." Do not, however, for a moment suppose that, in what I am saying, I am so ungracious as to wish to depreciate the value of class-examinations conducted by the professors themselves, such as those which have given occasion to the ceremony of to-day. These I hold to be of extensive and lasting benefit, and pray do not think me paradoxical when I add that they are perhaps more useful to those who fail than to those who succeed. The greater part of those who, in their subsequent career, attain high distinction, will be found to have been prizemen in their respective schools. But these are mostly men of genius, as somebody defined it, *i.e.*, men possessed of a great capacity for taking pains, men of that robust type of industry which would labour and succeed, prize or no prize. On the other hand, many who might otherwise remain sluggish and indolent are stimulated to action by the struggle for a prize, and the acquired habit of industry remains with them and is the greatest qualification for success. For the healing art, or rather so much of it as has yet been revealed to mankind, is not acquired once for all. In medicine, more than in most other professions, the labourer remains a student to the end, up to the close of his life in fact; for the pecuniary rewards of medicine rarely include more than a bare competence, a fair average share of the gifts of fortune, and partly on this account, and partly from the love of work and of usefulness, most of its votaries die in harness. But though your own experience, the proper digestion of whose teachings will, after you have quitted the school and the hospital, furnish your chief means of improvement—though, I say, your own laborious experience, and the necessary amount of medical reading to give you some idea of the experience of others, may leave you very little leisure, I would yet counsel you to bestow some part of that leisure upon some intellectual pursuit not too purely professional. It is good for almost every man to have what is irreverently called a hobby outside his ordinary occupation, as you will hereafter have occasion to observe among your patients; and it is good for doctor as well as for patient. The following, within the limits imposed by conscience and opportunity, of a scientific hobby, may have numerous advantages; for example, it may bring your mind into wholesome contact with those of superior men; but, after all, the great use of it is to enable you to withdraw your thoughts at times from the harassing and often grievous and depressing incidents of practice, and to escape, as it were, for a short while into a by-way or diverticulum, where the untrodden grass and flowers refresh you, and the thickets hide from you the gloomy incidents of daily life and daily struggle. From this point of view you are singularly fortunate in the nature of your academical education, and of the practical experience conjoined with the later stages thereof. Firstly, they give you a knowledge of man. Clergymen, it has been said, see men at their best, lawyers see them at their worst, physicians see them as they are; they see them affectionate or callous, sordid or magnanimous, patient or querulous, pure or sensual. Is it partly owing to this that so many of our most successful diplomatists have begun life as medical men—Crawford, for example, and Sir Rutherford Alcock and Sir John Kirk? Secondly, it tends to foster a kind, forbearing, and unselfish disposition; we are, it has been said, philanthropists by profession. Thirdly, it conducts you as far, at least, as to the threshold of several natural sciences, especially of the different branches of biology. Among these it is easy for a man to choose the one most congenial to his tastes or most convenient to his opportunities. In country districts the local practitioner is usually also the local geologist, botanist, and meteorologist; and in towns the expert microscopist may find some other branch of study in addition to that of normal histology or pathological anatomy. It is noteworthy how often side lights are reflected upon our work from these collateral sciences. I grant that the day when an Admirable Crichton was possible is long past, and that the extent and complexity of human knowledge is now so great that no man can hope to approach its boundaries except at one or two points. On the other hand, let me remind you that schoolmasters tell us how boys from the classical side often beat boys on the modern side in their own subjects; that the formulator of the law of Correlation of Forces was already a great lawyer, and now adorns the judicial bench; and that the late President of the Royal Society, whose loss we were lately so deeply deploring, was none the less able and thorough in his business as a printer because he shone so conspicuously in mathematics and in optics. Believe me, wide culture need not detract from practical ability, and narrowness is by no means always correlated with intensity.

### PRIZE LIST.

### SUMMER SESSION.

PRACTICAL CHEMISTRY.—*Prize:* C. J. Glasson. *Certificates:* W. J. Hill, H. F. Semple.

PRACTICAL PHYSIOLOGY AND HISTOLOGY.—*Prize:* A. J. Tomkins. *Certificates:* J. E. Trask, H. L. Evans, C. W. Lockyer, C. E. Dew, H. F. Semple, A. W. Riddell.

MATERICA MEDICA AND THERAPEUTICS.—*Prize:* T. Richards. *Certificates:* H. B. Densham, P. W. Menzies.

OBSTETRIC MEDICINE.—*Prize:* P. W. Williams. *Certificates:* W. G. Thorold, A. M. Gray, F. L. Davis, H. F. Parsons.

PRACTICAL SURGERY.—*Prize:* W. G. Thorold. *Certificates:* P. W. Williams, G. C. Helps.

OPERATIVE SURGERY.—*Prize:* W. J. T. Barker. *Certificates:* F. W. Weir, H. C. Thurston.

PATHOLOGY AND MORBID ANATOMY.—*Prize:* W. J. T. Barker.

### WINTER SESSION.

ANATOMY AND PHYSIOLOGY, JUNIOR CLASS.—*Prize:* W. M. Barclay.

JUNIOR CLASS OF ANATOMY.—*Lecturer's Prize:* A. H. Joseph. *Certificates:* H. F. Semple, W. J. Watkins, C. E. S. Flemming, C. T. Hudson, A. Leche.

JUNIOR CLASS OF PHYSIOLOGY.—*Certificates:* A. H. Joseph, C. E. S. Flemming, H. T. S. Aveline, T. R. Baker, H. A. Burleigh, R. F. W. Tucker, E. Ward, W. J. Watkins, H. F. Semple, C. T. Hudson.

SENIOR CLASS OF ANATOMY.—*Prize:* A. J. Tomkins. *Lecturer's Prize:* H. I. Pocock. *Certificates:* L. H. Williams, F. S. Hawkins, C. W. Lockyer, W. T. Ord, C. J. S. Shaw, J. B. Webb, C. J. Glasson, A. R. Aubrey, J. E. Trask.

Senior Class of Physiology.—*Prize:* A. J. Tomkins. *Certificates:* H. I. Pocock, H. L. Evans, L. H. Williams, A. W. Riddell, C. J. S. Shaw, W. C. Swayne, F. S. Hawkins, A. R. Aubrey, T. Richards, J. E. Trask.

Practical Anatomy.—*Prize:* L. H. Williams.

Chemistry.—*Prize:* W. M. Barclay. *Certificates:* E. Ward, H. T. S. Aveline, A. Leche, R. J. Marks, H. F. Semple, W. J. Watkins, G. Banbury.

Medicine.—*Prize:* A. N. Little. *Certificates:* W. J. T. Barker, W. G. Thorold, W. Basset.

Surgery.—*Prize:* W. G. Thorold. *Certificates:* A. N. Little, W. H. Stevens, W. Basset, P. W. Williams.

### BRISTOL ROYAL INFIRMARY.

Suple's Surgical Prize, a Gold Medal and Seven Guineas in Money.—W. G. Thorold.

Suple's Medical Prize, of the same value.—H. C. Thurston.

Tibbits' Memorial Prize, of the value of £7, for proficiency in Practical Surgery.—H. H. Tomkins.

Crosby Leonard Prize, for proficiency in Surgery.—H. H. Tomkins.

Pathological Prizes.—G. G. Adams, W. D. Duffett, H. C. Thurston.

### BRISTOL GENERAL HOSPITAL.

Clark Scholarship, of the value of £15, for proficiency in Surgery.—F. Morton and H. W. Windsor-Aubrey, equal.

Sanders' Scholarship, of the value of £22 10s., for proficiency in Medicine, Surgery, and Obstetric Medicine.—F. Morton.

Second Sanders' Scholarship, of the same value.—H. W. Windsor-Aubrey.

Lady Haberfield Prize, of the value of £30, for proficiency in Medicine, Surgery, and Obstetric Medicine.—W. J. T. Barker.

Pathological Clerkship Prize, of the value of £3 3s.—C. E. Evans.

---

Bristol General Hospital. *Winter Session, 1883—84.*—The Martyn Memorial Entrance Scholarship, of the value of £20, for proficiency in Classics, Mathematics, Modern Languages, and Physics, has been awarded to Mr. H. F. Devis.

